# Clinical Characteristics and Risk Factors of Mechanical Ventilation Among COVID-19 Patients on High-Flow Nasal Oxygen (HFNO)

**DOI:** 10.7759/cureus.65462

**Published:** 2024-07-26

**Authors:** Galal B Elrakaiby, Alaa E Ghabashi, Abdulrazak M Sakhakhni, Faris M Allaf, Saeed M Alamoudi, Muhammad A Khan

**Affiliations:** 1 Critical Care Medicine, King Abdullah International Medical Research Center, Jeddah, SAU; 2 Otolaryngology, King Abdullah International Medical Research Center, Jeddah, SAU; 3 Ophthalmology, King Faisal Specialist Hospital and Research Center, Riyadh, SAU; 4 Medical Education, King Saud Bin Abdulaziz University, Jeddah, SAU

**Keywords:** high-flow nasal cannula (hfnc), acute respiratory distress syndrome (ards), acute hypoxemic respiratory failure, covid-19 pneumonia, invasive mechanical ventilation, non-invasive mechanical ventilation, hfno, icu, viral pneumonia, covid-19

## Abstract

Introduction: COVID-19 is a viral infection affecting the respiratory system, primarily. It has spread globally ever since it first appeared in China in 2019. The use of high-flow nasal oxygen (HFNO) for the treatment of COVID-19 has not been well established.

Objectives: The primary objectives of this study are to observe the success of HFNO in preventing escalation to mechanical ventilation (MV) and to measure the prevalence of HFNO in King Abdulaziz Medical City (KAMC). The secondary objective is to describe patients who received HFNO clinically.

Methods: This is a retrospective cohort study of all polymerase chain reaction (PCR)-confirmed COVID-19 patients who require oxygen therapy in KAMC, Jeddah between March 1st, 2020, and December 31st, 2020. Any patients requiring MV on admission were excluded.

Results: 259 patients fit the inclusion criteria, and 25.5% of those included received HFNO. The number of non-survivors is 47 (18.1%). Mortality for HFNO, MV, and intensive care unit (ICU) are 30 (45.5%), 31 (60.8%), and 24 (32%), respectively. Their demographic was as follows; 160 were males, with a mean age of 60.93±15.01. Regarding the types of oxygen, low-flow nasal oxygen (LFNO) was administered to 243 out of the 259 patients, 66 received HFNO, 42 received MV, and 49 received other modes of ventilation. Additionally, 43.9% received HFNO escalated to MV. Patients who did not receive HFNO or MV were 178 (68.7%) in total.

Conclusion: The use of HFNO in COVID-19 patients could show better outcomes than MV in addition to preventing the use of MV. Larger studies are required to determine the efficacy of HFNO in COVID-19 patients.

## Introduction

In December 2019, Hubei Province, China witnessed an outbreak of an acute respiratory illness, currently known as COVID-19, which subsequently spread around the globe with over 45 million confirmed cases worldwide [1-3]. The spectrum of clinical presentation ranges from asymptomatic infection to acute respiratory distress syndrome (ARDS) and death [4]. Due to its status as a global pandemic, presentations of fever or recent contact with confirmed cases are regarded with a high index of suspicion for COVID-19, and molecular testing by reverse transcriptase-polymerase chain reaction (RT-PCR) acquired from the nasopharyngeal or oropharyngeal specimen is used to confirm or exclude the diagnosis [5,6]. Comorbidities are associated with a greater risk of severe disease and hospitalization as 91% of hospitalized patients in the US reported at least one preexisting medical condition [7,8]. The vast majority of patients experience mild febrile illness, yet a relatively high proportion require hospitalization and treatment with high-flow nasal oxygen (HFNO), non-invasive ventilation, or endotracheal intubation [4].

HFNO is a respiratory support modality that delivers high levels of heated and humidified oxygen through a nasal cannula at a precise fraction of inspired oxygen (FiO_2_) which assists in carbon dioxide (CO_2_) clearance and maintains low levels of positive pressure used in the treatment of hypoxemia [9,10]. Several invasive ventilation complications can be avoided by the use of HFNO such as ventilator-induced lung injury and nosocomial infections; however, it is an aerosol-producing modality with the risk of spreading infection [10]. On the other hand, HFNO is a useful treatment for ARDS to avoid intubation or as a bridge therapy without increased risk of mortality secondary to delayed intubation [10].

HFNO has been increasingly used for managing COVID-19 hypoxemic acute respiratory failure (hARF) unresponsive to conventional oxygen therapy [9]. It has been associated with lower intubation and mortality rates among COVID-19 hARF patients [2]. The literature review revealed no local studies in Saudi Arabia investigating the utility and outcomes of HFNO among COVID-19 patients. To bridge this gap, this study aims to determine the outcomes of HFNO as well as the clinical characteristics and comorbidities among hospitalized COVID-19 patients at King Abdulaziz Medical City, Jeddah.

## Materials and methods

Population 

The study includes all PCR-confirmed COVID-19 patients admitted to the hospital requiring oxygen therapy in KAMC, Jeddah between March 1, 2020, and December 31, 2020. Any patients fitting the previous description, but requiring mechanical ventilation (MV) on admission, were excluded. The study was approved by the King Abdullah International Medical Research Center (KAIMRC) (IRB: JED-21-427780-11804).

Study design and evaluation 

This is a retrospective cohort study. The sample size was calculated by using the Raosoft® software from the website www.raosoft.com/samplesize.html. The total number of COVID-19 patients admitted from March 1, 2020, to December 31, 2020, was estimated to be 120 patients. The required sample size was estimated at the 95% confidence interval level with an estimated 50% response distribution and a margin of error of ±5%. The required minimum sample size was determined to be 92. Although the required sample size is small, we elected to include all patients admitted to the hospital during the above-mentioned period with confirmed COVID-19 results.

Statistical analysis 

Data were collected and entered using a data collection sheet in Excel then transferred and analyzed using Statistical Package for the Social Sciences (SPSS version 20.0). The qualitative variables such as measured clinical outcomes and complication rates were presented as frequencies and percentages. Quantitative variables such as age, weight, and height were presented as mean ± standard deviation. The association between two categorical variables was assessed by the chi-square test. The association between numerical and categorical variables was assessed using the hypothesis test statistic (T-test)/analysis of variance (ANOVA) test. The association between two numerical variables was assessed by a correlation test. ANOVA test was used for the comparison between the three arms and a student t-test was used in the comparison between the two arms. A probability value (P-value) less than 0.05 was accepted for significance. 

## Results

In total, 259 patients fit the inclusion criteria out of 1263 COVID-19 patients. The incidence of patients who received HFNO was 66 (25.5%).

Oxygen therapy modalities

The incidence of oxygen therapy modalities received by patients at any time during their hospital stay is shown in Table 1. The most common modality was low-flow nasal oxygen (LFNO) administered to 243 (93.82%), followed by HFNO received by 66 (25.5%); nevertheless, patients commonly received more than one type of oxygen therapy throughout their hospital stay.

**Table 1:** Oxygen therapy modalities of the patients

Oxygen therapy modalities of the patients	
LFNO (1-5 L/min)	243 (93.82%)
HFNO (30-60 L/min)	66 (25.5%)
MV	51 (19.3%)
Non-rebreather mask	41 (15.83%)
Bilevel positive airway pressure	8 (3.09%)

Description of population

The mean ages of the patients were 60.93±15.0 years old. Other demographics and comorbidities are shown in Table 2, and Table 3 shows risk factors. 

**Table 2:** Demographic characteristics of the patients

Demographic characteristics of the patients	
Mean age (years)	60.93±15.01
Male (%)	160 (61.2%)
Female (%)	99 (38.2%)
Median (interquartile range) body mass index	30.2 kg/m^2^ (25.5-35.1 kg/m^2^)

**Table 3:** Risk factors of the patients HTN, hypertension

Risk factors of the patients	
HTN	54.4%
Diabetes	53.3%
Dyslipidemia	20.8%
Malignancy	16.2%
Ischemic heart disease	12.4%
Chronic kidney disease	8.9%
Asthma	8.9%
Immunosuppressive medications	6.6%
Heart failure	6.2%
Interstitial lung disease	3.1%
Chronic liver disease	2.3%
Chronic obstructive pulmonary disease	1.9%
Pulmonary HTN	1.2%

The clinical symptoms presented at the time of admission are illustrated in Table 4. Concerning vital signs documented at the time of presentation, 49 (18.92%) were hypoxemic on room air (O_2_ Sat <92%), 172 (66.41%) were tachypneic (respiratory rate >20), and 102 (39.38%) had an objective fever (T >38 C0). For HFNO patients, their vitals at the time of admission showed that 46 (69.7%) were tachypneic, and 22 (33.3%) were hypoxemic.

**Table 4:** Clinical symptoms of the patients

Clinical symptoms of the patients	
Shortness of breath	61%
Fever	50.2%
Dry cough	46.3%
Nausea/vomiting	20.5%
Diarrhea	18.9%
Productive cough	18.5%
Fatigue	18.1%
Headache	13.5%
Myalgia	11.2%
Anosmia	5.8%
Neurological symptoms	5.4%

Hospital course

At the time of initiation of oxygen therapy, chest imaging using X-ray or computerized tomography (CT) was obtained; the findings are shown in Table 5, and the major findings were as follows: 104 (40.15%) had bilateral peripheral infiltration, 90 (34.75%) had bilateral diffuse infiltration, and 39 (15%) had no infiltration.

**Table 5:** Chest imaging for patients

Chest imaging for patients		
	Bilateral (76.1%)	Unilateral (8.9%)
Peripheral	104 (40.15%)	16 (6.2)
Central	3 (1.2%)	0 (0%)
Diffuse	90 (34.75%)	7 (2.7%)
No infiltration	39 (15%)	

Concerning patients’ disposition, 75 (29%) required intensive care unit (ICU) admission, and 184 (71%) did not require ICU admission. Among the 66 patients who received HFNO, 54 (81.8%) were admitted to ICU. The remaining 12 HFNO patients (18.2%) remained in other disposition areas outside the ICU. Patients who did not receive HFNO or MV were 178 (68.7%) in total. Six (3.4%) out of 178 went to ICU. In addition, out of the 54 requiring HFNO and admitted to ICU, 36 (67%) had MV, and 18 (33%) did not have MV.

The median (interquartile range) length of stay (LOS) for our cohort is shown in Table 6; hospital LOS was 11 (7-19) days; ICU LOS was 8 (5-17.2) days. Additionally, the LOS of survivors was 10 (7-17.2) days, and the LOS of non-survivor patients was 17 (10-28.2) days. 

**Table 6:** LOS LOS, length of stay

LOS		
	Median	Interquartile range
Hospital	11	(7-19)
ICU	8	(5-17.2)
Survivors	10	(7-17.2)
Non-survivors	17	(10-28.2)

Outcomes

The relationship between HFNO and MV is shown in Table 7. Among patients who received HFNO, 30 (45.5%) patients did not escalate to MV and 36 (54.5%) escalated to MV; in addition, the remaining patients who escalated to MV were 15 (7.7%). The median (IQR) duration of HFNO use was two days (one to five days). 

**Table 7:** The relationship between HFNO and MV for patients HFNO, high-flow nasal oxygen; MV, mechanical ventilation

The relationship of HFNO and MV for patients				
	MV			
HFNO	Yes	No	Total	
Yes	36 (54.5%)	30 (45.5%)	66 (25.5%)	
No	15 (7.7%)	178 (92.3%)	193 (74.5%)	
Total	208 (80.7%)	51 (19.3%)	259	

The overall outcomes of the patients are shown in Table 8. The number of non-survivors is 47 (18.1%) and the number of survivors is 212 (81.9%). Regarding the overall mortality for HFNO, MV, and ICU, the results are 30 (45.5%), 31 (60.8%), and 24 (32%), respectively. The mortality of HFNO admitted to ICU patients is 26 (48.15%); the mortality of MV escalated from HFNO patients is 24 (67%), and the mortality number for HFNO who did not escalate to MV is three (16.7%). Finally, the mortality for those HFNO patients who remained outside the ICU is four out of 12 (33.3%). 

**Table 8:** Mortality of patients (% out of specific population) HFNO, high-flow nasal oxygen; MV, mechanical ventilation; ICU, intensive care unit

Mortality of patients (% out of specific population)	
Overall for HFNO patients	30 (45.5%)
Overall for MV patients	31 (60.8%)
HFNO escalated to MV patients	24 (67%)
HFNO patients who were admitted to ICU and did not escalate to MV	3 (16.7%)
HFNO patients who remained in ward	4 (33.3%)
ICU admitted patients with no HFNO or MV	0
Ward patients who did not receive HFNO or MV	9 (5.2%)

## Discussion

In this retrospective single-center cohort study of patients requiring supplementary oxygen due to COVID-19 infection, HFNO was used for 66 (25.5%) patients. This finding is closer to the 19% reported by the COVID-ICU Group in a multicenter study including 4643 subjects; on the other hand, a higher incidence rate was reported by Bonnet et al., where the rate of HFNO use was 55% of 138 patients [11,12]. Our study shows similar results compared to the COVID-ICU Group but lower in comparison to Bonnet et al. The lower application rate of HFNO in our study could be explained by the fact that our cohort included patients admitted to both the general wards and ICU; hence, the majority of our cohort had relatively mild severity of COVID-19 disease; on the other hand, Bonnet et al. and the COVID-ICU Group designed their study to include only ICU patients [11,12].

The use of HFNO may potentially halt the need for escalation to MV. In a randomized controlled trial published by Ospina-Tascón et al., the use of HFNO was shown to be effective in significantly decreasing the need for MV with only 34.3% of the patients receiving HFNO escalating to MV within the first 28 days of admission [13]. In our study, the percentage of patients requiring MV after receiving HFNO was 36 (54.5%). The higher escalation percentage in our study could be due to delayed administration of HFNO among our patients compared to Ospina-Tascón et al. where the patients were assigned to their treatment arm on admission [13]. However, Bonnet et al. reported a closer percentage of 39 out of 76 (51%) of escalation to MV [12]. 

The mean age in our study population is 60.93±15.01. Closer results are reported by Patel et al., COVID ICU group, Bonnet et al., and Ospina-Tascón et al., where they reported 60.66 (±13.50), 63 (54-71), 59 (48-67), and a median age of 60, respectively [2,11-13]. The similar age group throughout these studies could point out the increased COVID-19 severity in this age group. 

The prevalence of hypertension (HTN) in the Saudi population reported by Alhabib et al. is 30.3% in adults aged 46.5±9.12 years old; diabetes mellitus (DM) is reported with an incidence of 18.7% in the general Saudi population by the International Diabetes Federation [14,15]. The most common comorbidities among the patients included in our study were HTN and DM, 54.4% and 53%, respectively. Of note, the rate of HTN and DM in our cohort is higher than in the general population. This could point to the fact that pre-existing HTN or DM correlated with an increase in disease severity. This finding is consistent with Liu et al.’s study of COVID-19 patients [7].

The two most common presenting symptoms in our cohort are shortness of breath (61%) and fever (50.2%). According to Li et al., the most common symptoms were fever (78.8%), followed by cough (53.9%) [16]. These dissimilar findings between the two studies could be attributed to the study design where we included only hospitalized patients requiring oxygen treatment, where the main presenting symptom was shortness of breath; however, in the meta-analysis by Li et al. both out-patients and in-patients were included [16].

Regarding imaging studies, the majority of patients had bilateral lung infiltrates (197, 76.1%). Lu et al.’s study among adults found that 72.3% of patients confirmed to have COVID-19 had bilateral opacities [17]. 

Regarding patients’ disposition, 75 (29%) required ICU admission, and 184 (71%) did not require ICU admission. Our research shows a close ICU admission rate to Chang et al. reporting a pooled ICU admission rate of 21% [18]. 

With regards to the LOS, hospital LOS in our study was 11 (7-19) days. Ospina-Tascón et al. reported LOS of 14 (9-23) days [13]. For patients admitted to the ICU, the LOS is predictably longer; our study reported a relatively lower LOS of eight (5-17.2) days; on the other hand, the COVID-ICU Group reported ICU LOS of 16 (9-28) days [11].

Regarding medication, our study result for antibiotics use is 251 (96,6%). As reported by Chedid et al., the mean rate of antibiotic use was 74.0% of cases in nineteen clinical studies [19]. These results may be explained by the usual administration of antibiotics as prophylactic empiric treatment for superimposed bacterial pneumonia or other conditions such as infiltration or signs of infection. However, according to the National Institute of Health, no clinical trials have evaluated the use of antibiotics in patients with COVID-19, and there is insufficient evidence to recommend either for or against antibiotic therapy in COVID-19 patients in the absence of other causes [20]. In our study, corticosteroids were used in 211 (81.5%). Ospina-Tascón et al. reported systemic steroids used in 93 patients (93.9%) of the HFNO group vs. 92 (92.0%) of the conventional oxygen therapy group [13]. Nevertheless, the National Institute of Health recommends Dexamethasone initiation in all COVID-19 patients requiring oxygen [20]. The remaining patients in our study didn’t receive corticosteroids because corticosteroids were not accepted as a standard of care at the beginning of the pandemic. Anticoagulants were used in 214 (82.6%) patients. The National Institute of Health recommends a prophylactic dose of heparin in patients requiring LFNO, HFNO, and ICU admission, and following anticoagulant indications by the usual standard of care for patients without COVID-19 [20]. The remaining patients who did not receive anticoagulants in our study were ambulatory or had contraindications to use anticoagulants. 

The number of non-survivors in our study is 47 (18.1%) in all patients and 24 (32%) in ICU patients. COVID-ICU Group reported an overall mortality of 31% and a decreased mortality from 42% to 25% throughout the study period for ICU-admitted patients [11]. Moreover, Ospina-Tascón et al. found a non-significant mortality difference between HFNO and standard oxygen therapy groups (12% vs 24%; OR 0.52 (95%CI, 0.2-1.34) p= 0.17 and 16% vs 26%; OR 0.75 (95%CI, 0.32-1.8) p=0.52, respectively) [13]. This could be related to the fact that HFNO is applied later in the more severe stage of COVID-19 disease and to ICU-admitted patients similar to the COVID-ICU Group and Ospina-Tascón et al. [11,13]. 

## Conclusions

The use of HFNO may potentially decrease the need for escalation to MV and improve outcomes among admitted COVID-19 patients requiring oxygen supplementation. Patients who are older, diabetic, and/or hypertensive are compromised for more severe COVID-19 disease. Our study showed that lower LOS could be a prognostic marker for a favorable survival outcome in the COVID-19 population. Further prospective studies to assess the utility of HFNO in preventing escalation to MV are warranted.
